# Verification of Vision-Based Terrain-Referenced Navigation Using the Iterative Closest Point Algorithm Through Flight Testing

**DOI:** 10.3390/s25185813

**Published:** 2025-09-17

**Authors:** Taeyun Kim, Seongho Nam, Hyungsub Lee, Juhyun Oh

**Affiliations:** 4th R&D Institute, Agency for Defense Development, Daejeon 34060, Republic of Korea; shnam@add.re.kr (S.N.); suby0913@add.re.kr (H.L.); juhyunoh@add.re.kr (J.O.)

**Keywords:** terrain-referenced navigation, vision sensor, stereo matching, iterative closest point algorithm

## Abstract

Terrain-referenced navigation (TRN) provides an alternative navigation method for environments with limited GPS availability. This paper proposes a vision-based TRN framework that employs stereo imagery and a rotation-invariant iterative closest point (ICP) algorithm to align reconstructed elevation maps with a terrain elevation database. In contrast to conventional ICP, which is sensitive to camera intrinsic errors, the proposed approach improves robustness at high altitudes. Its feasibility and effectiveness are demonstrated through full-scale flight tests using a Cessna aircraft equipped with an IMU, camera, and barometric altimeter. The results show that the proposed method consistently enhances positioning accuracy and robustness compared with a filter-based approach, particularly under challenging high-altitude conditions where image resolution is reduced. The algorithm proved capable of maintaining reliable performance across varying flight altitudes, demonstrating its robustness under high-altitude conditions. This study establishes the novelty of integrating rotation-invariant ICP with vision-based TRN and provides real-world validation through actual flight testing. The findings offer valuable implications for future research and potential applications in unmanned aerial vehicles and long-range guided systems, where passive and GPS-independent navigation is critical for mission success.

## 1. Introduction

In typical outdoor environments, integrated GPS/INS (Global Positioning System/Inertial Navigation System) systems are commonly utilized for the navigation of both manned and unmanned aircraft. However, in certain weapon systems such as guided missiles and drones, the use of GPS can be severely restricted due to electronic interference such as intentional jamming or spoofing. In these GPS-denied scenarios, terrain-referenced navigation (TRN) serves as a representative and practical alternative. TRN offers a significant advantage in that it does not depend on external signals or aids. Instead, it enhances inertial navigation accuracy by reducing drift errors through the comparison of measured terrain elevations against stored terrain elevation databases, such as the Digital Terrain Elevation Data (DTED) and Digital Elevation Models (DEM) [1,2,3]. One of the earliest and most influential TRN techniques is the Terrain Contour Matching (TERCOM) algorithm [4], originally developed in the 1970s for cruise missiles and aircraft prior to the widespread deployment of GPS. With the integration of Kalman filtering and advances in computational power, TERCOM evolved into more sophisticated systems such as the Sandia Inertial Terrain-Aided Navigation (SITAN) system [5,6] and the Terrain Profile Matching (TERPROM) system [7].

Traditionally, TRN systems rely on radio altimeters to obtain terrain elevation measurements [8,9,10,11,12]. However, as an alternative for platforms that are difficult to mount a radio altimeter, various studies have recently been conducted on TRN using an image sensor embedded in the platform [13,14,15,16,17]. An image sensor has the limitation that it can measure the distance through an indirect method based on stereo analysis and is affected by weather conditions. Nevertheless, unlike a radio altimeter, which must emit radio waves to the ground, an image sensor can operate in a passive manner to avoid detection by the enemy in military operations. Vision-based navigation techniques have been actively investigated for aerial vehicles [18,19,20]. Ref. [21] demonstrated an approach that integrates vision and inertial data to improve visual odometry performance, validated through full-scale helicopter experiments. Ref. [22] proposed enhancements for stereo visual odometry tailored for high-altitude operations. Additionally, various map-referenced navigation methods that leverage satellite or aerial imagery databases have been proposed [23,24,25,26,27]. Vision-based map-referenced navigation algorithm that performs the terrain classification through semantic segmentation using Fully Convolutional Networks (FCN) was suggested in [28].

Vision-based TRN methods commonly estimate or correct positions by directly inputting terrain elevations derived from stereo images into filtering algorithms. However, this paper proposes an alternative approach that leverages the ICP algorithm to obtain position fixes by aligning stereo-derived elevation maps with reference databases. By using the transformation output of the ICP, rather than the raw terrain elevations, as the measurement model, the proposed method improves filter robustness and mitigates divergence risks caused by low-resolution or inaccurate maps.

The proposed vision-based TRN system estimates a vehicle’s position by matching altitudes extracted from aerial imagery with a terrain elevation database. Three-dimensional elevation information is obtained through stereo matching sequential aerial images captured by a downward-facing image sensor. The resulting terrain map is then aligned with the reference database via the ICP algorithm to yield a position fix, which is subsequently used as an input to a filtering algorithm for position estimation. The method follows a three-step process: image rectification, elevation map generation, and position estimation.

First, an image pair is selected from the continuously acquired aerial images, ensuring adequate baseline distance for effective stereo matching. Rectification is performed to align image scan lines, using projection matrices derived from the camera’s intrinsic and extrinsic parameters. In the elevation map generation step, stereo matching is applied to the rectified images to find correspondences and compute disparity maps over the overlapping image region. The 3D terrain map is then reconstructed using the disparity map, the baseline between images, and the camera’s altitude, with baseline values derived from the extrinsic parameters obtained via inertial navigation. During the final step, the ICP algorithm is used to register the generated terrain map to the reference database by estimating a transformation. This transformation is used as a position fix input to the TRN filter. Residual checks are performed to prevent incorrect position updates based on ICP errors.

To validate the feasibility and accuracy of the proposed method, flight tests were conducted using a Cessna aircraft equipped with both an IMU and a camera. The vision-based TRN was performed using the inertial navigation results and aerial images captured throughout the flight. The test results were analyzed to assess the performance of each component: image rectification, elevation map generation, and position estimation.

This study extends the authors’ previous work [29], which utilized a standard ICP implementation and was validated through Monte Carlo simulations. In addition, Navon et al. [30] employed a conventional ICP algorithm to align camera-derived point clouds with a digital terrain model (DTM), demonstrating the feasibility of vision-based TRN through simulation studies and emphasizing robustness with outlier filtering.

In contrast to these simulation-based evaluations, the present work demonstrates a complete onboard implementation involving real sensors—including an IMU, a camera, and a barometric altimeter—mounted on an aircraft. The proposed algorithm is further enhanced by employing a rotation-invariant ICP to improve robustness against intrinsic parameter errors of the camera, which are particularly detrimental at high altitudes. Its feasibility is rigorously validated through actual flight tests using a Cessna aircraft. This combination of methodological improvement and experimental validation establishes the novelty and practical significance of our approach.

## 2. The Proposed Vision-Based Terrain-Referenced Navigation

**Figure 1:**
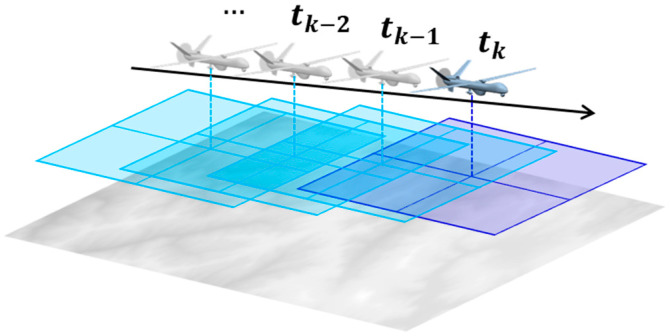
The configuration of acquisition of aerial images for vision-based TRN.

The proposed method continuously captures aerial images using a downward-facing image sensor mounted on the vehicle, as illustrated in Figure 1. From the sequence of acquired images, two images are selected based on the ratio of the baseline length to the vehicle’s operational altitude, since a sufficient baseline is essential for accurate terrain elevation extraction via stereo matching. In the selected pair, the earlier and later images are treated as the left and right images of a stereo system, respectively. A 3D terrain elevation map is generated from the image pair and then compared with a reference terrain elevation database to estimate the vehicle’s position.

**Figure 2:**
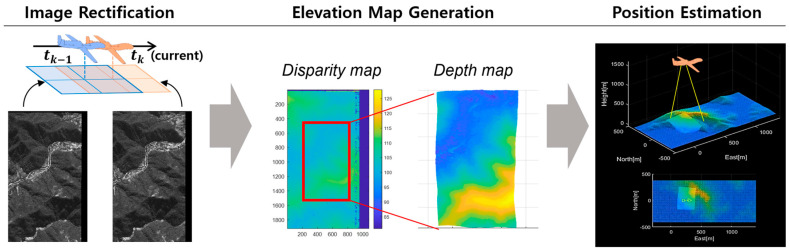
A schematic of vision-based TRN which consists of three steps: image rectification, elevation map generation, and position estimation.

The entire process consists of three primary steps: image rectification, elevation map generation, and position estimation. Figure 2 illustrates the overall procedure.

### 2.1. IMU Kinematics

The change in camera pose between the two images is derived from the inertial navigation result computed using the measurements from the onboard IMU. This device measures the angular rate of the sensor with respect to an inertial frame in the body frame, and specific force in the body frame. The measurements are given as follows.

(1) $${\tilde{f}}^{b}=f^{b}+b^{a}+n^{a}\;{\tilde{\omega }}_{ib}^{b}=\omega_{ib}^{b}+b^{g}+n^{g}$$

Here, ${\tilde{f}}^{b}$ and ${\tilde{\omega }}_{ib}^{b}$ are accelerometer measurements and gyroscope measurements, and $f^{b}$ and $\omega_{ib}^{b}$ are the true specific force and true angular rate, respectively. $b^{a}$ and $b^{g}$ are the accelerometer and gyroscope biases. $n^{a}$ and $n^{g}$ are the accelerometer and gyroscope noise.

The IMU kinematics model in the navigation frame is given as below [31].

(2) $${\dot{R}}_{b}^{n}=R_{b}^{n}{\left[\omega_{ib}^{b}\right]}_{\times }-{\left[\omega_{in}^{n}\right]}_{\times }R_{b}^{n}\;{\dot{v}}^{n}=C_{b}^{n}f^{b}-({\left[\omega_{en}^{n}\right]}_{\times }+2{\left[\omega_{ie}^{n}\right]}_{\times })v^{n}+g^{n}\;{\dot{p}}^{n}=M_{pv}v^{n}$$

$R_{b}^{n}$ denote the rotation matrix from the body frame to the navigation frame, $v^{n}={\left[v_{e}\;\;v_{n}\;\;v_{u}\right]}^{T}$ is the velocity in the navigation frame, $p^{n}={\left[L\;\;\lambda \;\;h\right]}^{T}$ is the absolute position represented by latitude, longitude and height. $\omega_{in}^{n}$ is the angular rate of the navigation frame with respect to the inertial frame, $\omega_{ie}^{n}$ is the earth rate, and $\omega_{en}^{n}$ is the transport rate. The dot notation denotes the time derivative of the corresponding variable, while ${\left[\cdot \right]}_{\times }$ represents the skew-symmetric matrix form of a 3D vector used to compute the cross product in matrix form. The matrix $M_{pv}$ is given by

(3) $$M_{pv}=\left[\begin{array}{ccc} 0 & \frac{1}{R_{M}+h} & 0\\ \frac{sec\;L}{R_{N}+h} & 0 & 0\\ 0 & 0 & 1 \end{array}\right]$$

where $R_{N}$ and $R_{M}$ are the meridian radii of curvature and the transverse radii of curvature.

### 2.2. Image Rectification

**Figure 3:**
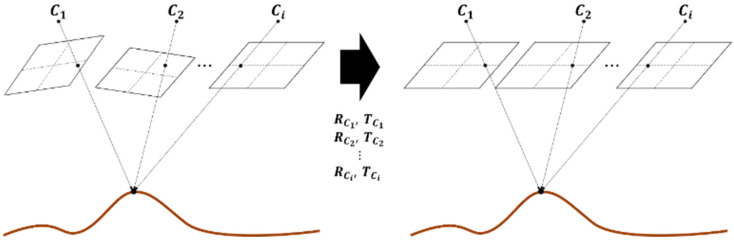
Image rectification based on the result of inertial navigation.

Image rectification transforms the selected image pair into a format with aligned epipolar lines, so that disparity occurs only along the horizontal axis (x-direction), as depicted in Figure 3. To perform rectification, the projection matrix of each image is computed using the camera’s intrinsic and extrinsic parameters. Intrinsic parameters are obtained via calibration, while extrinsic parameters are derived from the inertial navigation result at the time of image capture.

The equation of the projection matrix is as below.


(4) $$\lambda \left[\begin{array}{c} u\\ v\\ 1 \end{array}\right]=K\left[R|T\right]\left[\begin{array}{c} X_{w}\\ Y_{w}\\ Z_{w}\\ 1 \end{array}\right]$$

**Figure 4:**
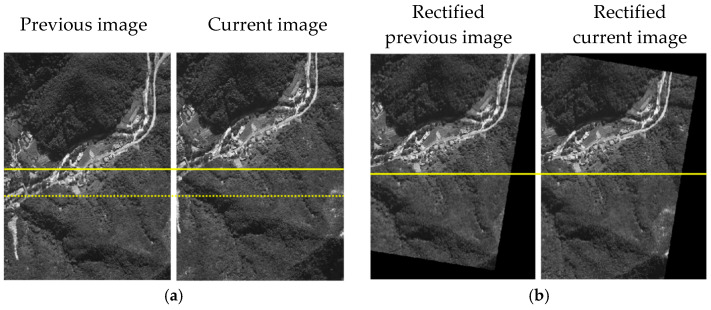
Image rectification result. (**a**) aerial image set; (**b**) rectified aerial image set.

Here, u and v are pixel coordinates in the camera frame and $X_{w}$, $Y_{w}$, and $Z_{w}$ are three-dimensional points in the world frame. λ is non-zero scalar representing the projective depth. K represents the camera intrinsic matrix and R and T are the rotation matrix and translation vector, respectively. $\left[R|T\right]$ denotes the camera extrinsic matrix and $K\left[R|T\right]$ is the projection matrix. Since R and T are calculated using the inertial navigation result, they include the inertial navigation drift error. Each image is projected through the obtained projection matrix so that the images are represented in the same plane. Figure 4 shows the result of the image rectification.

### 2.3. Elevation Map Generation

With the rectified image pair, the semi-global matching (SGM) algorithm [32] is applied to identify pixel correspondences and calculate disparities across the entire overlapping region. Disparity computation can be affected by various types of errors, including rectification inaccuracies and image distortions, which may introduce outliers into the disparity map. To mitigate this, a linear search adjusts the position of the previous image in the direction perpendicular to the scan lines to minimize outliers, yielding a corrected disparity map.

The disparity between the correspondence pixels is related to the camera’s focal length, f, baseline, B, and depth of the object, z by the following relationship:

(5) $$disparity=u-u^{’}=\frac{Bf}{z}$$

**Figure 5:**
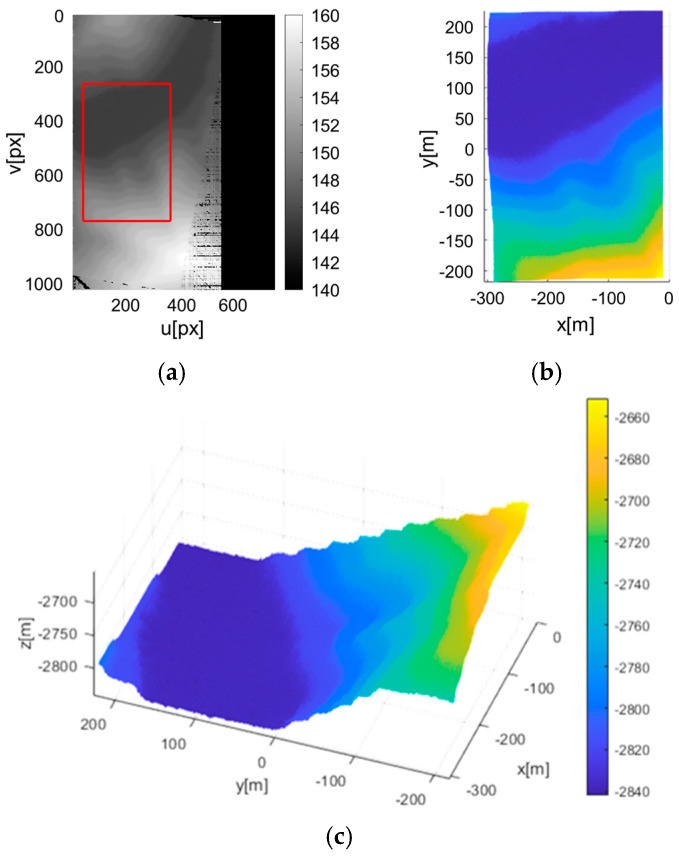
Elevation map generation result. The red line indicates the area of the elevation map. (**a**) disparity map; (**b**) 2D depth map; (**c**) 3D depth map.

Based on the above equation, the three-dimensional terrain elevation map corresponding to the disparity map can be generated using the given focal length and baseline between the images. The baseline is derived from the extrinsic parameters obtained via inertial navigation. Figure 5 presents the disparity and corresponding depth maps. The stepped appearance of the depth map results from quantization limits. According to Equation (5), the altitude resolution per pixel disparity is governed by the focal length and baseline. Similarly, the elevation accuracy can be expressed as

(6) $$\sigma_{z}=\frac{z^{2}}{Bf}\sigma_{px}$$

where $\sigma_{z}$ and $\sigma_{px}$ denote the elevation accuracy and the disparity accuracy.

(7) $$f=\frac{w}{2}{\left[tan\frac{\theta }{2}\right]}^{-1}$$

The focal length in pixels can be expressed as a function of the image size, w and the field-of-view (FOV), θ as shown in Equation (7). According to these relationships, increasing the baseline and narrowing the FOV improve elevation accuracy but may also introduce challenges: longer baselines increase navigation drift errors, while smaller FOVs reduce image overlap and increase sensitivity to attitude errors. Thus, the sensor configuration must balance these trade-offs based on the operational altitude and navigation system performance.

**Figure 6:**
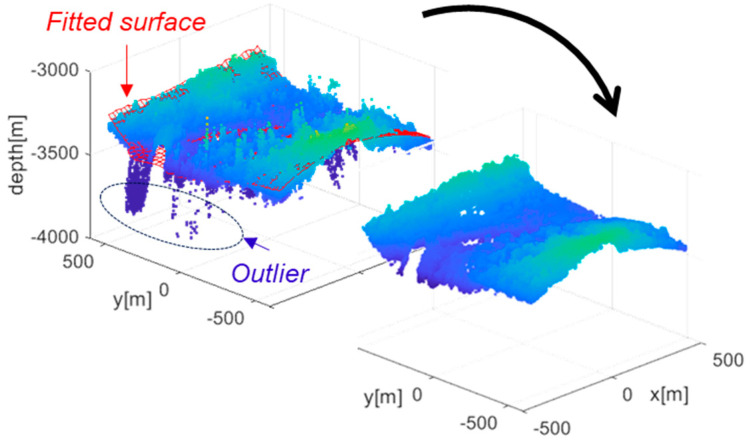
Removal of outliers using surface fitting.

During depth map generation, outliers may arise from image noise or correspondence errors. Since terrain can be reasonably modeled as a continuous surface, points that deviate significantly from a locally fitted surface to the depth map can be identified outliers. To eliminate spurious measurements, a polynomial surface fitting approach is employed. A second-third order bivariate polynomial surface is fitted to the map points, and the Euclidean distance between each point and the fitted surface is computed. Points with distances greater than a predefined threshold are classified as outliers and excluded from subsequent analysis. The threshold is determined such that it exceeds natural terrain variation, i.e., abrupt altitude differences on the order of several tens of meters that cannot occur in continuous terrain. Removing these outliers helps reduce depth map errors and improve overall map quality (see Figure 6).

### 2.4. Position Estimation

In the position estimation step, the vehicle’s position is estimated by aligning the generated terrain elevation map with a reference database. This alignment is achieved using the ICP algorithm, which computes a transformation that minimizes the distance between two 3D point clouds. In this context, one point cloud is the elevation map derived from aerial imagery, and the other is the terrain database.

While standard ICP [33] optimizes both rotation and translation, our proposed method employs a rotation-invariant ICP, which estimates only translation. In high-altitude imagery, the large distance between the camera and the terrain surface degrades the reliability of rotation estimation within the ICP framework. To address this limitation, we assume that the elevation map, generated from vertically rectified nadir imagery, shares the same orientation as the terrain database. Accordingly, the rotation matrix is fixed to the identity, and only translation is estimated, resulting in more robust and reliable registration performance. To further mitigate potential errors caused by residual tilt in the rectification process, the ICP results are incorporated as measurements in a filtering algorithm, which is then used for position estimation. This approach allows the filter to handle occasional poor ICP results due to rectification inaccuracies, thereby improving the robustness and accuracy of the estimated positions.

In Algorithm 1, designate point cloud A as the reference database and point cloud B as the generated map, then compute the translation between them. In this study, the initial transformation $T_{0}$ is set as the previous navigation estimate from the TRN. A point-to-point Euclidean distance metric is employed, and nearest neighbor search is performed using a k-d tree. All correspondences are used for alignment. The ICP iteration is capped at 30 iterations, with a stopping tolerance of 0.01 for the change in mean squared error. These settings are consistently applied across all ICP-based experiments to ensure reproducibility.
**Algorithm 1** Rotation-invariant ICP**Input**
 Two point clouds: $A=\left\{a_{i}\right\}$, $B=\left\{b_{i}\right\}$
 An initial translation: $T_{0}$,
 A constrained rotation: R=I.**Output**
The correct translation, T, which aligns A and B
**Procedure**
 $T\leftarrow T_{0}$
 **while** not converged do
    **for** i←1 to N do
       $m_{i}\leftarrow FindClosestPointInA\;(I{\cdot b}_{i}+T)$;
       **if**  $\left\|m_{i}-(I{\cdot b}_{i}+T)\right\|\leq d_{max}$ then
          $w_{i}\leftarrow 1$;
       **else**
          $w_{i}\leftarrow 0$;
       **end**
    **end**
    $T\leftarrow \underset{T}{\arg\min}\left\{\sum_{i}w_{i}{\left\|(I{\cdot b}_{i}+T)-\;m_{i}\right\|}^{2}\right\}$;
  **End**

**Figure 7:**
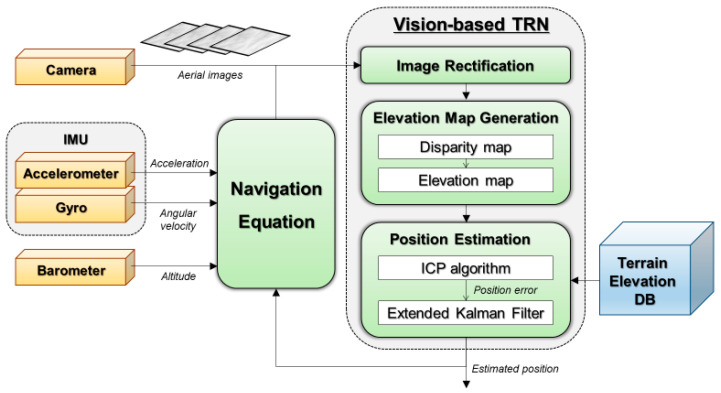
Structure of vision-based TRN with ICP.

The resulting translation vector from ICP is treated as a position fix and used as the measurement input for an Extended Kalman Filter (EKF) [34,35]. The rotation-invariant ICP provides full 3-D translation estimates $\left[t_{x},\;t_{y},\;t_{z}\right]$, but only the horizontal components $\left[t_{x},\;t_{y}\right]$ are incorporated as measurements in the EKF. Because the camera captures terrain from relatively high altitudes, the vertical component $t_{z}$ is highly sensitive to small errors in the camera focal length, which often leads to a vertical bias. Consequently, the $t_{z}$ from ICP is not used for correcting barometric drift, as it is considered less reliable than the barometric altimeter under these conditions. Figure 7 illustrates the overall structure of the vision-based TRN system incorporating ICP. The system and measurement models used in the EKF are as follows.

(8) $$x={\left[x\;\;y\right]}^{T}\;x_{k}=x_{k-1}+u_{k-1}+w_{k-1}\;z_{k}=x_{k}+v_{k}$$

Here, x and y represent the horizontal position of the vehicle, and u is the inertial navigation-based position increment. This measurement model uses the position fixes from the ICP as the measurements, $z_{k}$. w and v are the process noise and the measurement noise which are assumed to be a zero-mean Gaussian distribution. The covariance of the process noise reflects the performance of inertial navigation, which is determined by the characteristics of the IMU. The covariance of the measurement noise indicates the accuracy of the position fixes obtained from ICP and is influenced by the precision of the generated map.

Due to the error of the generated map or the terrain shape of the database, ICP may converge wrong, resulting in incorrect results. To prevent incorrect updates, a residual check is performed for each ICP measurement by comparing the measurement residual with the estimate covariance propagated from both the measurement and process noise. In this study, measurements exceeding the 3-sigma range of the estimate covariance are rejected and not used for filter updating, ensuring robust and reliable position estimation.

Moreover, during flight segments with rapid attitude changes, image overlap is reduced, which can degrade disparity computation and map generation. In addition, even when the roll or pitch angles are relatively large, the nadir images may exhibit reduced overlap during the rectification process, similarly affecting depth map quality. Consequently, position updates for these segments are excluded to maintain estimation accuracy.

## 3. Flight Test Results

**Figure 8:**
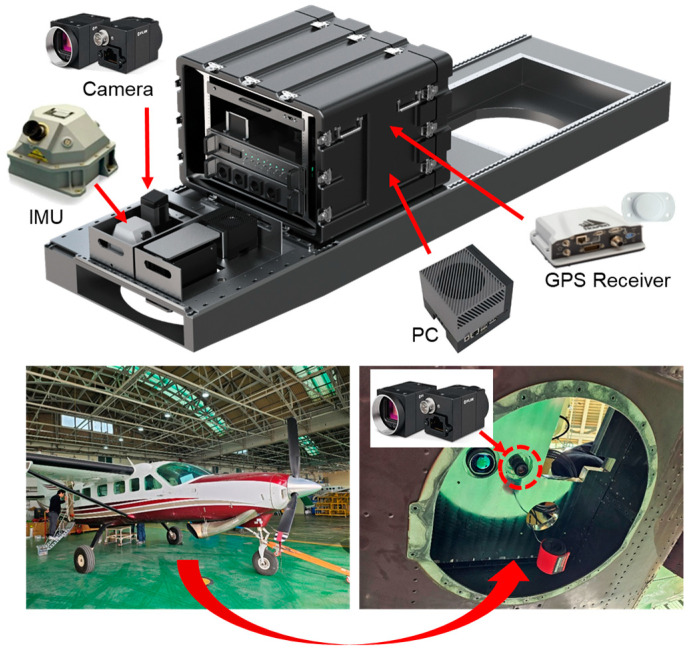
Equipment for the aerial flight test.

To evaluate the performance of the proposed vision-based TRN algorithm, a flight test was carried out using a Cessna aircraft equipped with an IMU and a downward-facing camera, as shown in Figure 8. The reference trajectory for performance evaluation was obtained using GPS data. The vision-based TRN was performed using the inertial navigation results based on IMU data and the continuously captured aerial images throughout the flight.

**Figure 9:**
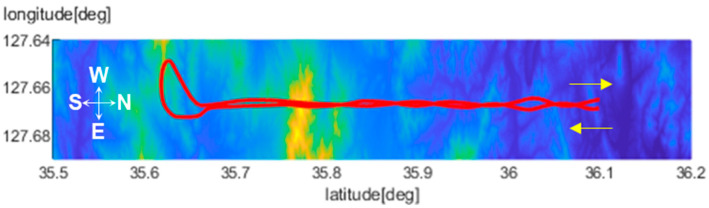
Flight test trajectory.

Figure 9 illustrates the flight trajectory. The aircraft first traveled southward, completed a 360-degree turn, and returned to its original starting position. Tests were conducted at altitudes of 3 km, 4 km, and 5 km, with one complete round-trip performed at each level. The DEM used for ICP alignment in this study is a DTED Level 4 dataset with 5 m posting, referenced to mean sea level (MSL) and using the GRS80 coordinate system. The DEM covers the area surrounding Wanju-gun, Republic of Korea. Prior to performing ICP, camera and INS heights were converted to the same datum to ensure accurate alignment.

Table 1 summarizes the flight conditions and sensor specifications. To ensure a minimum 80% overlap between successive images, image pairs were selected to maintain a baseline of at least 120 m. The baseline distance was determined considering flight speed, altitude, and the camera’s field of view (FOV). The nominal flight speed was approximately 90 m/s, and the total flight duration was 1200 s. Position estimates were updated at a frequency of 1 Hz.

The camera used in our experiments is a FLIR Blackfly S (Teledyne FLIR LLC, Wilsonville, OR, USA), featuring a 1/1.1-inch CMOS sensor with a pixel pitch of 2.74 μm and a global shutter. Lens distortion parameters were calibrated as k1 = −0.0760, k2 = 0.2329, k3 = −0.0245, p1 = −1.749 × 10^−4^, and p2 = −2.901 × 10^−4^, resulting in a reprojection RMS of 0.192 px. Time synchronization between the camera, IMU, and barometer was experimentally measured to ensure consistent temporal alignment of all sensor data. The camera mounting geometry was determined according to a custom-designed rig, with its actual dimensions directly measured and applied during all flight tests.

The barometric altitude was initialized using the International Standard Atmosphere (ISA) method. Its bias was estimated through the integrated GPS/INS filter prior to entering the flight test area. To ensure consistency with the DEM, barometric measurements were converted to the MSL vertical datum.

**Figure 10:**
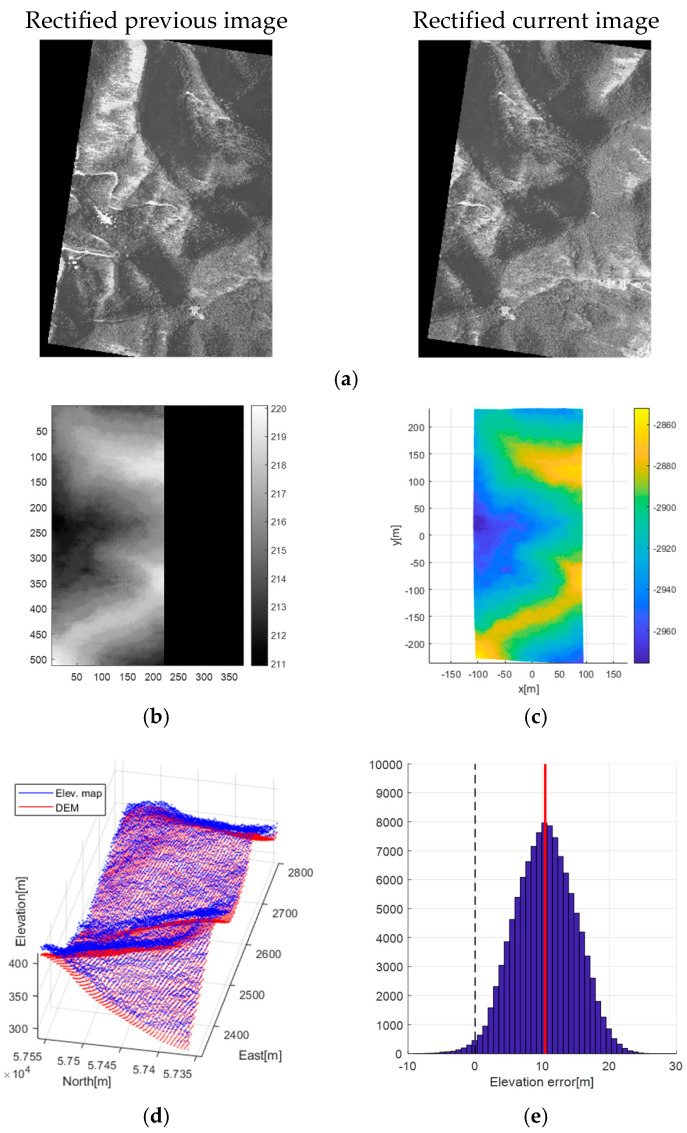
Result of image rectification and elevation map generation during the flight test. (**a**) aerial images; (**b**) disparity map; (**c**) elevation map; (**d**) map comparison with DEM; (**e**) elevation error histogram.

Figure 10 presents the results of the image rectification and elevation map generation processes. The two images used in the example were taken 2 s apart to ensure an appropriate baseline. From these, a disparity map was generated using the SGM algorithm, and the corresponding elevation map was reconstructed using the baseline derived from the inertial navigation results. The parameter settings for the SGM algorithm are shown in Table 2. The generated elevation map was compared against the DEM of the same area to evaluate its accuracy, and it was observed that the elevation error follows a Gaussian distribution. Figure 10d shows a side-by-side comparison of the generated elevation map (blue point cloud) and the reference DEM (red point cloud), revealing strong visual similarity. However, the error histogram in Figure 10e shows a vertical bias. As noted in Section 2.4, this bias arises from reconstruction errors caused by the camera capturing terrain from relatively high altitudes, making it sensitive even to small focal length inaccuracies.

**Figure 11:**
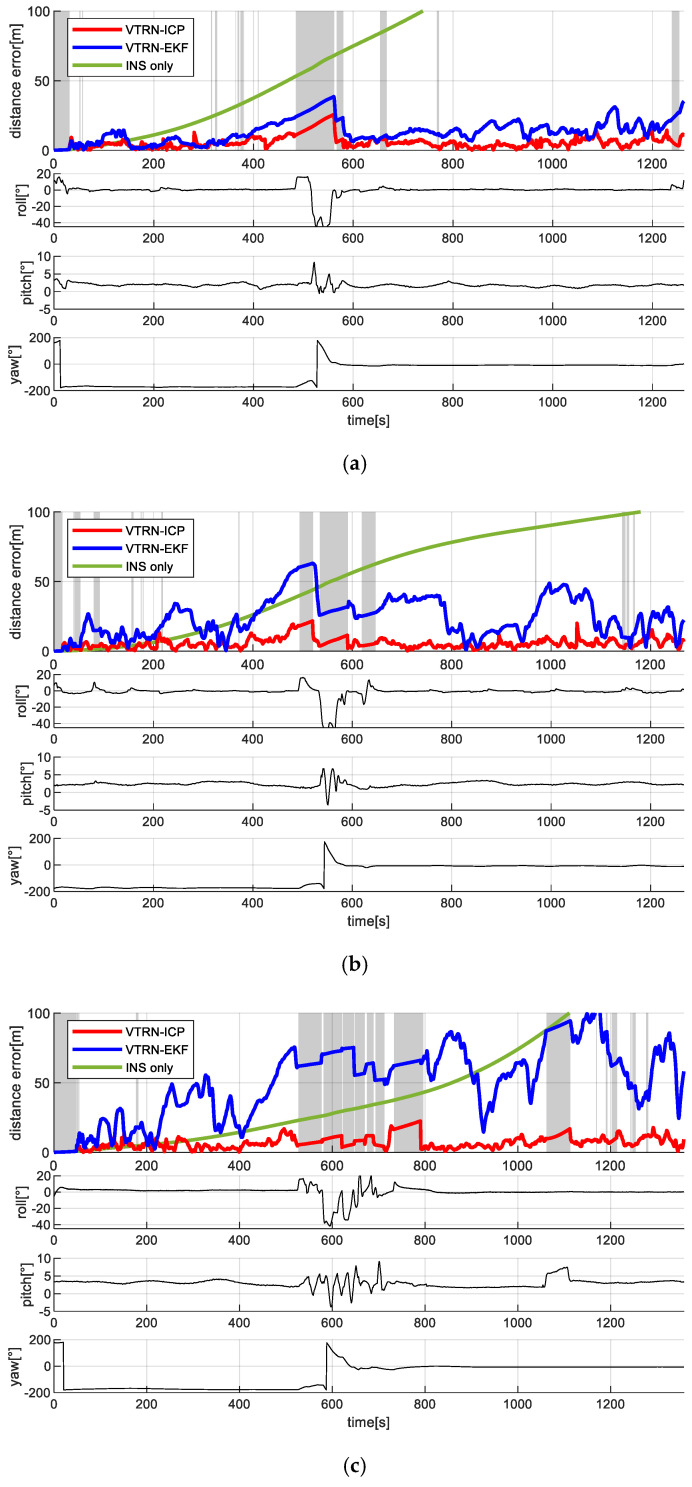
Time trajectories of the distance error along the flight altitudes. (**a**) 3000 m altitude; (**b**) 4000 m altitude; (**c**) 5000 m altitude.

To validate the effectiveness of the ICP-based TRN method, its position estimation results were compared with those of a conventional Kalman filter-based approach that directly incorporates terrain elevation measurements. Figure 11 shows the distance errors for each of the three test altitudes (3000 m, 4000 m, and 5000 m). Gray-shaded regions in the plot indicate periods of rapid attitude change or large roll/pitch angles during flight, during which position updates were suspended and the navigation relied solely on inertial data, resulting in gradual error growth. While the Kalman filter method showed acceptable accuracy at lower altitudes (e.g., 3000 m), its performance degraded noticeably as the altitude increased. This degradation was due to the reduced accuracy of the elevation maps at higher altitudes, which increases the likelihood of incorrect convergence. In contrast, the proposed ICP-based method consistently maintained reliable navigation accuracy across all altitudes, demonstrating its robustness.

**Figure 12:**
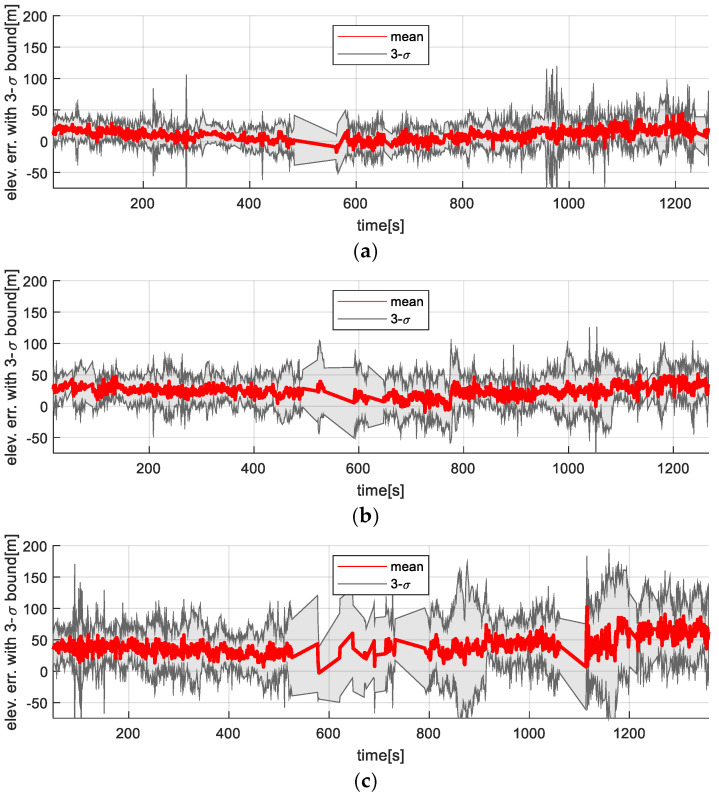
Vision-based TRN with KF result: Time trajectories of the generated map error along the flight altitudes. (**a**) 3000 m altitude; (**b**) 4000 m altitude; (**c**) 5000 m altitude.

**Figure 13:**
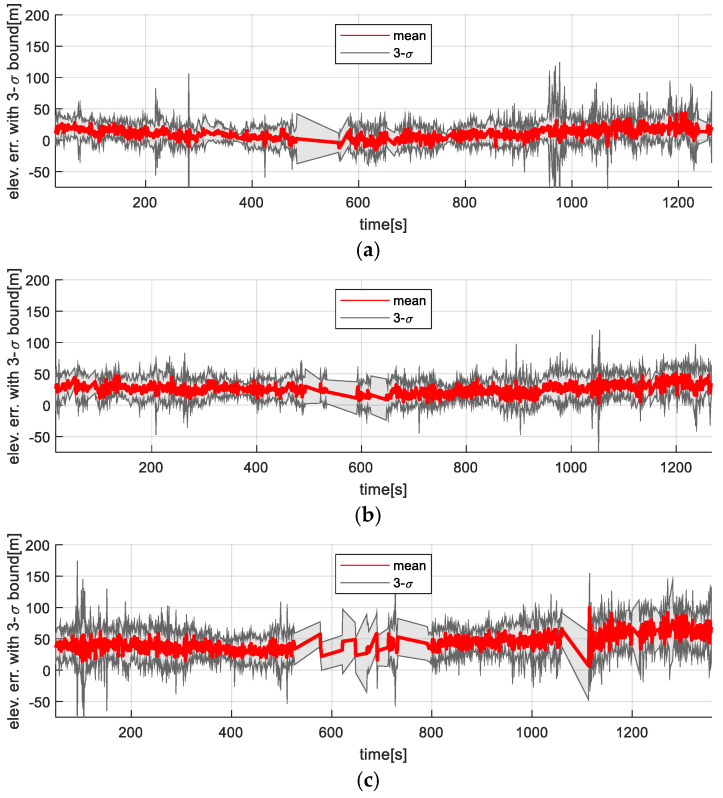
Vision-based TRN with ICP result: Time trajectories of the generated map error along the flight altitudes. (**a**) 3000 m altitude; (**b**) 4000 m altitude; (**c**) 5000 m altitude.

Figure 12 and Figure 13 show the time-series variation in the elevation map error statistics for the filter-based TRN method and the ICP-based TRN, respectively. As expected, the accuracy of the generated maps decreased with altitude. While the filter-based method is directly affected by this degradation (since elevation is used as a direct measurement), the rotation-invariant ICP method remains more resilient, as it leverages relative shape alignment between the generated map and the reference DEM rather than relying on absolute height values.

**Figure 14:**
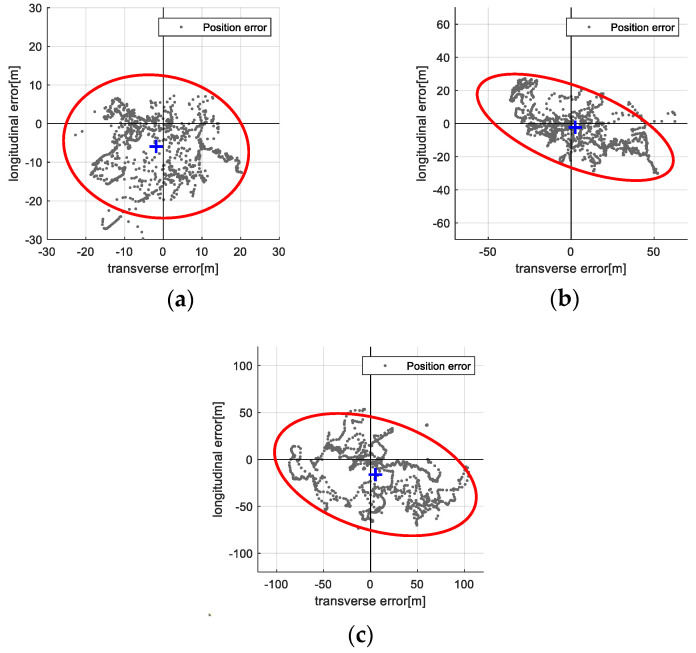
Vision-based TRN with KF result: Position error distribution on the body axis with 95% confidence ellipse. (**a**) 3000 m altitude; (**b**) 4000 m altitude; (**c**) 5000 m altitude.

**Figure 15:**
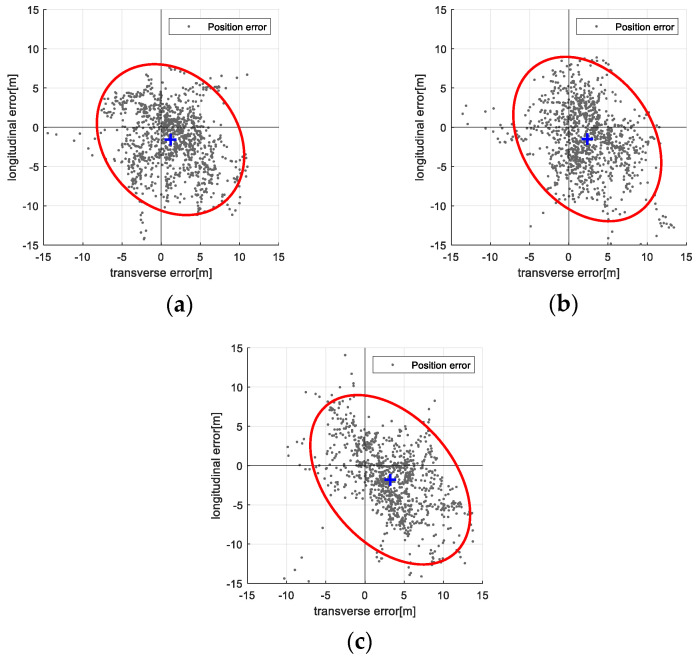
Vision-based TRN with ICP result: Position error distribution on the body axis with 95% confidence ellipse. (**a**) 3000 m altitude; (**b**) 4000 m altitude; (**c**) 5000 m altitude.

Table 3 presents statistical summaries (mean and standard deviation) of navigation and map errors for both the filter-based TRN and ICP-based TRN methods at each test altitude. While errors generally increase with altitude, the ICP-based method consistently outperforms the filter-based method in both navigation accuracy and map quality. Figure 14 and Figure 15 visualize the distribution of position errors in the vehicle’s body frame using 95% confidence ellipses: Figure 14 for the filter-based method and Figure 15 for the ICP-based method. The ICP-based method exhibits smaller error spreads and tighter clustering around the true position, providing clear quantitative evidence of its performance advantage and validating the proposed approach.

## 4. Conclusions

This paper introduced a vision-based TRN method that estimates a vehicle’s position by aligning altitudes extracted from aerial images with a known terrain elevation database. The approach utilizes stereo matching of sequential aerial imagery captured by a downward-facing camera to reconstruct three-dimensional terrain elevation maps. These maps are then aligned with a reference database using the ICP algorithm to produce position fixes, which serve as inputs to a filtering algorithm for final position estimation.

The proposed system demonstrates reliable performance even in high-altitude scenarios, where traditional TRN methods relying on radio altimeters or direct elevation measurements may struggle due to reduced resolution or increased noise. By applying image rectification and leveraging inertial navigation outputs to enhance stereo matching, the system effectively reconstructs terrain maps across varying altitudes. To address the difficulty of rotation estimation in high-altitude imagery, a rotation-invariant ICP was adopted, focusing solely on translation optimization.

The method was validated through flight tests using a Cessna aircraft equipped with an IMU and camera. The results confirm the effectiveness of the approach by comparing it with conventional Kalman filter-based methods. While the Kalman filter performed well at lower altitudes, its accuracy declined with increasing altitude due to elevation map inaccuracies. In contrast, the proposed ICP-based TRN method consistently maintained high accuracy and robustness regardless of altitude.

In summary, the proposed vision-based TRN framework using rotation-invariant ICP provides a promising and practical alternative for reliable navigation in GPS-denied environments, particularly in aerial applications operating at high altitudes.

It should be noted, however, that the method assumes modest inter-frame attitude differences due to the short temporal separation (≈1–2 s) between stereo images and the nadir-looking imaging geometry. Even when using an IMU of lower grade than the navigation-grade unit employed in our tests, the resulting orientation drift over such short intervals is typically limited, and the ICP performance degrades gradually rather than failing catastrophically. Residual-based gating further prevents faulty updates from entering the filter. Thus, while large and rapid attitude changes could reduce map overlap and impact registration accuracy, under the tested conditions the proposed approach remained robust and effective.

The performance of TRN is also fundamentally dependent on terrain configuration. In flat terrain, horizontal position updates cannot be reliably obtained, which is an inherent limitation of TRN. However, in the high-altitude setting considered in this work, the camera captures a wide area in a single stereo pair, such that the reconstructed terrain exhibits greater variability across the broader scene. This allows the proposed method to be more effective than approaches relying on small, localized terrain patches, even when local terrain elevation variations are modest.

Future research may explore several directions to further enhance the robustness and practicality of the proposed system in real-world scenarios. One potential area involves developing a precise in-flight calibration technique to accurately estimate and correct the misalignment between the onboard inertial navigation system and the camera. Another important consideration is improving the system’s ability to maintain reliable navigation performance during aggressive maneuvers, where rapid attitude changes can reduce image overlap and disrupt disparity computation. Additionally, alternative approaches could be investigated for use in cloudy or visually degraded environments, where terrain features may be partially or fully occluded due to adverse weather conditions.

## Figures and Tables

**Table 1:** Parameters for vision-based TRN flight test.

**Camera Specification**	
Sensor model	FLIR-BFS-PGE-120S6C-C
Focal length	5974.82 [px]
Image size	4096×3000 [px]
Horizontal and Vertical FOVs	37.84, 28.19 [deg]
**IMU specification**	
Sensor model	Honeywell HG9900
Acceleration bias error	25.0 [μg]
Gyro bias error	0.004 [deg/hr]
Gyro angular random walk	$0.002\;[deg/\sqrt{hr}]$
**Barometer specification**	
Sensor model	Honeywell HPA200
Accuracy	±0.03% FS Max.
Operating range	0~17.6 [psia]
**Nominal speed**	90 [m/s]
**Nominal altitude**	3000, 4000, 5000 [m]

**Table 2:** Parameter settings for SGM algorithm.

Parameter	
Matching cost	Census transform
Window size	9 × 7 [px]
Number of aggregation paths	8
Penalty parameters (P1 and P2)	10 (P1), 120 (P2)
Disparity range	0~192 [px]
Uniqueness ratio	0.95
Sub-pixel method	Quadratic interpolation
Left-right consistency check	Enabled
Median filtering	3 × 3 [px]
Hardware	CPU (Optimized with SSE and OpenMP)
Image downsampling	4×

**Table 3:** Navigation and map error statistics from vision-based TRN flight test.

	Test Altitude [m]		3000	4000	5000
**Navigation error [m]**	KF-based	Mean			
		Transverse axis	−1.86	2.48	5.17
		Longitudinal axis	−5.92	−2.24	−16.16
		Standard deviation			
		Major axis	9.83	25.76	45.41
		Minor axis	7.52	9.67	23.93
	ICP-based	Mean			
		Transverse axis	1.19	2.35	3.22
		Longitudinal axis	−1.58	−1.50	−1.80
		Standard deviation			
		Major axis	4.28	4.66	5.10
		Minor axis	3.44	3.38	3.29
**Map error [m]**	KF-based	Mean	9.91	23.81	40.97
		Standard deviation	11.85	14.05	22.91
	ICP-based	Mean	10.53	25.80	44.22
		Standard deviation	10.96	10.64	16.53

## Data Availability

Data are contained within the article.
